# Genotype-Phenotype Correlation in Patients with Congenital Adrenal Hyperplasia due to 21-Hydroxylase Deficiency in Cuba

**DOI:** 10.1155/2021/9316284

**Published:** 2021-01-06

**Authors:** Tania Mayvel Espinosa Reyes, Teresa Collazo Mesa, Paulina Arasely Lantigua Cruz, Adriana Agramonte Machado, Emma Domínguez Alonso, Henrik Falhammar

**Affiliations:** ^1^National Institute of Endocrinology, Zapata Street and D. Vedado, Havana, Cuba; ^2^National Center for Medical Genetics, Havana, Cuba; ^3^Department of Molecular Medicine and Surgery, Karolinska Institutet, Stockholm, Sweden; ^4^Department of Endocrinology, Metabolism and Diabetes, Karolinska University Hospital, Stockholm, Sweden

## Abstract

**Background:**

There are several studies that show a good genotype-phenotype correlation in congenital adrenal hyperplasia (CAH) due to 21-hydroxylase deficiency (21OHD). However, there is well-documented evidence of inconsistency in some cases.

**Objectives:**

To determine if there is a correlation between the identified mutations and the clinical manifestations of 21OHD in the Cuban population.

**Methods:**

A cross-sectional descriptive study of all patients referred for a molecular diagnosis of 21OHD in Cuba from January 2000 to December 2018. The clinical manifestations of each patient were identified and classified according to the phenotype. The *CYP*21*A*2 gene was analyzed for the presence of 5 point mutations involved in the pathogenesis of 21OHD (intron 2, deletion of 8bp, I172N, P30L, and Q318X); correlation was sought between the phenotypic characteristics and the frequencies of point mutations in the patients using the Spearman test.

**Results:**

A total of 55 patients underwent direct analysis of the *CYP*21*A*2 gene in order to determine the presence of the 5 point mutations. Point mutations were identified in 31 patients, which corresponded to 56%. A statistically significant genotype-phenotype correlation was found.

**Conclusions:**

The correlation between the detected molecular defect and the clinical expression of 21OHD was reasonable in the Cuban population, which could allow phenotypic predictions to be made from the genotype.

## 1. Introduction

Congenital adrenal hyperplasia due to the deficiency of 21-hydroxylase (21OHD) is an autosomal recessive disease caused by mutations in the *CYP*21*A*2 gene located on the short arm of chromosome 6. Between 65 and 70% of patients are compound heterozygotes, and the clinical expression is the result of the mildest mutation [1, 2].

According to the degree of the 21-hydroxylase enzymatic activity, 3 different phenotypes can be distinguished: the classic salt-wasting (SW), the classic simple virilizing (SV), and the nonclassical form (NC) [3, 4].

The classic forms constitute the most serious forms, and the girls present with variable virilized external genitals at birth but with normal internal genitals [4]. Scrotal hyperpigmentation and macrogenitosomy may appear in males. Mineralocorticoid deficiency, most pronounced in the SW form, can cause severe hyponatremia, hyperkalemia, and acidosis that, in the absence of adequate treatment, may be fatal or be more insidious, with asthenia anorexia and poor weight gain [5]. In the SV form, the girls present with a variable spectrum of genital ambiguity, while in the boys, the diagnosis is more difficult and is often made due to precocious pseudopuberty with growth rate acceleration and skeletal maturation [4, 5]. However, in countries with neonatal screening, the diagnosis of classic 21OHD is often picked up by the screening program [6].

Patients with the NC phenotype show signs of hyperandrogenism with acceleration of growth velocity, bone age, early pubarche, acne, hirsutism, and/or menstrual disorders. Most are diagnosed in young adulthood, but many are probably never diagnosed, especially the males [7].

Hundreds of *CYP*21*A*2 mutations have been found to be involved in the pathophysiology of 21OHD [8]. Conversions and deletions are responsible for a high percentage. Other mechanisms such as uniparental disomy and de novo mutations have also been described [1, 2, 9].

There are several studies that have shown a good genotype-phenotype correlation. However, there are well-documented inconsistencies in some cases [10–14].

In Cuba, the genotype-phenotype correlation in patients with CAH due to 21OHD has not been studied previously. Thus, this was the aim of the present study.

## 2. Materials and Methods

This was a descriptive cross-sectional and observational study of all patients referred for a molecular diagnosis of 21OHD in Cuba from January 2000 to December 2018. Referrals came from different pediatric departments all over the country. First, the clinical manifestations of each patient were identified, and, based on these elements, they were classified according to clinical forms of presentation. The diagnosis had been confirmed with measurements of elevated levels of 17-hydroxyprogesterone in all cases. Secondly, the mutational analysis of the *CYP*21*A*2 gene was performed in order to identify point mutations involved in the pathogenesis of 21OHD. In this study, only point mutations derived from the pseudogene (*CYP*21*A*1) were analyzed, specifically P30L‚ intron 2‚ 8bp deletion, I172N, and Q318X. Exon 1 (g.89C > *T* (p.P30L)) is a mild missense single base pair mutation associated with 21-hydroxylase enzyme activity between 30 and 60% [12, 15]. Intron 2 (g.655C/*A* > *G*) is associated with aberrant splicing due to upstream activation of a splice acceptor site and an enzyme activity of less than 2–5% [1, 2, 9]. Exon 3, 8bp deletion (g.707_714delGAGACTAC (p.G110fs)), is a frame shift mutation associated with premature codon termination and complete loss of enzyme activity [9]. Exon 4 (g.999*T* > *A* (p.I172N)) is associated with loss of hydrophobic package and reduction of enzyme activity to 2–10% [1, 2, 9]. Exon 8 mutations (g.1994C > *T* (p.Q318X) and g.2108C > *T* (p.R356W)) are associated with disruption of H-bonding and loss of 21-hydroxylase enzyme [1, 9].

To detect the association between phenotypic characteristics and the frequencies of point mutations in the patients, Chi-square tests were performed. A value of *p* < 0.05 was considered statistically significant.

## 3. Results

A total of 55 patients underwent direct analysis of the *CYP*21*A*2 gene in order to determine the presence of the 5 point mutations. The mean ages at biochemical and clinical diagnosis were for patients with SW 13.4 ± 6.3 days, for SV 12.8 ± 3.4 months, and for NC 13.6 ± 3.7 years, respectively.

**Figure 1:**
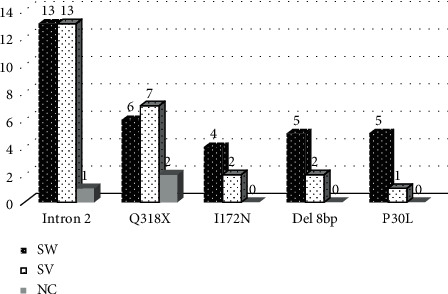
Alleles affected by mutations according to the clinical forms of 21-hydroxylase deficiency in the Cuban population.

Point mutations were identified in 31 patients, which corresponded to 56%.  Figure 1 shows the number of alleles affected by mutations, according to phenotype.

The phenotype-genotype correlation of the studied patients is shown in Table 1.

When performing a Chi-Square test, a statistically significant association was found between the clinical manifestations and the identified point mutations (*p*=0.024). With the purpose of trying to homogenize the groups, the variable detected mutations were reorganized depending on the number of mutations identified (2, 3, 4, or more). When analyzing this new variable and its relationship with the clinical forms of presentation, statistical significance was also obtained (*p*=0.000).

## 4. Discussion

This is the first genotype-phenotype correlation study done on the Cuban population. We found a reasonable correlation, and these data add to the increasing number of studies on genotype-phenotype correlation in 21OHD [10–12].

It is important to know to what degree a certain genotype can predict which phenotype a patient will have [6]. In the case of 21OHD, as there are many different mutations, the number of combinations is high, and since a certain allele can have more than one mutation, the number of possible combinations is even higher.

In recessive diseases, it is necessary that each of the two alleles present at least one mutation, although these may be different mutations. Since not all mutations affect the enzymatic activity of 21-hydroxylase equally, the severity of the disease will, in theory, be determined by the mutation that least affects the enzyme activity [16]. The difference between the clinical forms of 21OHD will therefore depend on the differences in the degree of enzyme activity, and this will be the result of a specific mutation.

The classic form of CAH is the result of the presence of two severely affected alleles, while in the NC form, one or two mildly affected alleles or one severe and one mildly affected alleles are present. The latter is known as compound heterozygotes.

In the current study, a high correlation was observed between the intron 2 and exon 8 Q318X mutations with the classic forms. However, in 3 subjects, their presence in heterozygosis resulted in a NC phenotype. It is important to remember that the 10 most frequent *CYP*21*A*2 mutations cause variable effects on the phenotype and do not always agree with the genotype [17]. In addition, when the genotype-phenotype correlation is explored in patients with homozygous mutations, the correlation is close to 100%. In contrast, the predictive capacity of the phenotype is less when there are different mutations on each allele [18]. This explains much of the variability of clinical expression in the studied patients, where 10 subjects presented mutations in compound heterozygosis.

In the great majority of patients who presented combinations of two serious mutations, the SW form was established, and in the majority of patients with two mild mutations, the NC form was demonstrated. The genotypes with the worst phenotype correlation were those with a severe mutation together with an intermediate or milder mutation (intron 2/I172N and 8bp/I172N deletion, respectively) [17].

Finkielstain et al. [18] showed in 1986 that there was genotype-phenotype agreement as high as 90.5% for SW, 85.1% for SV, and 97.8% for NC. However, in 2013, New et al. [11] in an investigation involving 1,507 subjects with CAH only found a direct correlation in 46.7% of the genotypes studied.

It is important to emphasize that, in the patients with the SW form, even though the presence of the mutations justifies the clinical form, the distribution of the mutations in each allele cannot be specified since that would require a complete gene sequencing. Discrepancies between genotype and phenotype can be of two types. First, the genotype predicts a milder condition than that observed phenotypically. Second, the genotype predicts a more severe entity than that is observed phenotypically. The first type of discrepancy can be due to the presence of more than one mutation in the same allele, while the second type, the more complex to explain, has been attributed to the presence of mutations in intron 2, due to variable levels of enzyme activity associated with this mutation [10, 19, 20].

The P30L mutation is produced by a change in position 69 of cytokine by thymine in exon 1 of the gene, causing instability in proline, an amino acid that largely forms a species of stem in which the enzyme is anchored in microsomal membrane. When this mutation occurs, the orientation of 21-hydroxylase in the membrane changes, and its activity decreases up to 30–60% [15]. This moderate activity would justify its association with a mild form. However, P30L mutation has, up to 30%, been associated with classic 21OHD, especially if intron 2 mutation was found on the other allele [21]. This coincided with the result of the present study.

The homozygosity of intron 2 mutations and its association with the SV form has been described less frequently than with the SW form [10, 11, 21–24], although some have found the opposite [25, 26]. The intron 2 mutation is produced by a replacement of cytosine/adenine by guanine at position 655 that creates an aberrant splice site by removing 19 nucleotides from the mRNA. This results in a protein that generally conserves between 2 and 5% of its enzymatic activity, which would explain the greater association with SW [21, 24, 25, 27], which was confirmed in the current study.

The 8-base pair deletion mutation (Del8bp) consists of the deletion of 8 base pairs in exon 3, which originates a truncated protein with no enzymatic activity. It is widely associated with SW forms, and in our study, it behaves similarly [24, 27]. It is important to note that there is no history of inbreeding in the families studied, so it can be assumed that, in the Cuban population, the carrier frequency of the various mutations is high.

The Q318X mutation occurs by substitution of cytosine for thymine at position 1994, and it is associated with disruption of the H ligand and total loss of the synthesis of the 21-hydroxylase enzyme. Its presence is associated with the classic form [28]. In the current study, it appeared in 7 (23%) of the patients, and in 5 of them, it was associated with severe phenotypes, similar to what has been described in the literature [29]. In 3 of the patients, it was found in compound heterozygosis.

The I172N mutation is produced by a substitution of thymine for adenine at position 999, associated with the loss of the hydrophobic region and a 2–10% reduction in the 21-hydroxylase activity. The I172N mutation is present in 25% of all classic forms, especially SV [29, 30]. In the patients studied, it was present in 3 of them, in compound heterozygosis (I172N, Q318X), and indeed associated with SV form. However, in 2 patients with I172N in homozygosis, it was associated with the SW form.

Thus, a detailed characterization of the patients is possible, and with it, a clearer interpretation from a pathophysiological point of view is obtained. This allows establishing better strategies for follow-up, a more accurate prognosis, and the possibility of establishing more precise genetic counseling [30]. Moreover, there are some indications that genotype may also predict long-term outcomes [31–33].

The limitations are (1) the limited number of included patients and (2) *CYP*21*A*2-specific PCR, followed by sequencing, combined with multiplex ligation-dependent probe amplification (MLPA) or real-time PCR for quantitation was not available due to economical constraints. A full gene sequencing and a familial genetic segregation analysis ideally should have been performed to be able to study and explain in detail some lack of concordance of genotype-phenotype correlation in these patients. However, our study shows the reality in countries with economical constraints.

## 5. Conclusions

The correlation between the detected molecular defect and the clinical expression of 21OHD in Cuban patients with CAH was reasonable, allowing possible phenotypic predictions to be made from the genotype. Also, these findings should assist physicians in prenatal diagnosis and genetic counseling of parents who are at risk for having a child with 21OHD.

## Figures and Tables

**Table 1:** Genotype-phenotype correlation in Cuban patients with 21-hydroxylase deficiency.

Molecular findings			Patients, their clinical forms, and gender									
			P2	P3	P4	P5	P6	P7	P12	P15	P16	P17
			SW	SW	SW	SW	SW	SW	SW	SW	SW	SW
			F	F	F	M	M^∗^	F	F	F	F	M

P30L		Heterozygous	x									
		Homozygous										
Intron 2		Heterozygous	x			x						
		Homozygous		x	x			x			x	x
Del8bp		Heterozygous	x				x					
		Homozygous										
Q318X		Heterozygous										
		Homozygous							x	x		
I172N		Heterozygous										
		Homozygous	x									

Molecular findings			Patients, their clinical forms, and gender									
			P20	P21	P22	P23	P24	P25	P28	P29	P30	P33
			SW	SW	SW	SW	SW	SW	SV	SV	SV	SV
			F	F	F	F	F	F	M	M	F	F

P30L	Heterozygous											
	Homozygous											
Intron 2	Heterozygous									x	x	
	Homozygous		x		x				x			x
Del8bp	Heterozygous							x				
	Homozygous											
Q318X	Heterozygous											
	Homozygous					x	x					
I172N	Heterozygous											
	Homozygous			x								

Molecular findings			Patients and their clinical forms									
			P35	P36	P39	P40	P44	P45	P46	P47	P48	P49
			SV	SV	SV	SV	SV	SV	SV	NC	NC	NC
			F	F	F	F	F	M^∗^	M	F	F	F

P30L	Heterozygous											
	Homozygous											
Intron 2	Heterozygous		x	x				x	x			x
	Homozygous											
Del8bp	Heterozygous					x						
	Homozygous											
Q318X	Heterozygous									x	x	
	Homozygous				x		x					
I172N	Heterozygous											
	Homozygous											

^*∗*^46XX. SW, salt-wasting phenotype. SV, simple virilizing phenotype. ^*∗*^46XX. SV, simple virilizing phenotype. NC, nonclassic phenotype.

## Data Availability

The datasets generated and/or analyzed during the current study are not publicly available because it belongs to the National Institute of Endocrinology, but are available from the corresponding author on reasonable request.
